# Intranasal delivery of phenytoin loaded layered double hydroxide nanoparticles improves therapeutic effect on epileptic seizures

**DOI:** 10.1186/s12951-024-02405-8

**Published:** 2024-04-02

**Authors:** Jingxin Zhang, Huali Zuo, Yanlu Fu, Yina Cao, Qiwei Li, Qi Zhang, Yuyi Zheng, Yi Wang, Di Wu, Weiyu Chen, Jiajia Fang

**Affiliations:** 1 Department of Neurology, The Fourth Affiliated Hospital, Zhejiang University School of Medicine, Yiwu, 322000 China; 2 The Fourth Affiliated Hospital, Zhejiang University School of Medicine, Yiwu, 322000 China; 3 Key Laboratory of Neuropharmacology and Translational Medicine of Zhejiang Province, School of Pharmaceutical Sciences, Zhejiang Chinese Medical University, Hangzhou, 310053 China; 4 Department of Respiratory and Critical Care Medicine, The Fourth Affiliated Hospital, Zhejiang University School of Medicine, Yiwu, 322000 China

**Keywords:** Epilepsy, Phenytoin, Intranasal delivery, Layered double hydroxide nanoparticles

## Abstract

Improving the efficiency of antiseizure medication entering the brain is the key to reducing its peripheral toxicity. A combination of intranasal administration and nanomedicine presents a practical approach for treating epileptic seizures via bypassing the blood-brain barrier. In this study, phenytoin (PHT) loaded layered double hydroxide nanoparticles (BSA-LDHs-PHT) were fabricated via a coprecipitation − hydrothermal method for epileptic seizure control. In this study, we expound on the preparation method and characterization of BSA-LDHs-PHT. In-vitro drug release experiment shows both rapid and continuous drug release from BSA-LDHs-PHT, which is crucial for acute seizure control and chronic epilepsy therapy. In-vivo biodistribution assays after intranasal administration indicate excellent brain targeting ability of BSA-LDHs. Compared to BSA-Cyanine5.5, BSA-LDHs-Cyanine5.5 were associated with a higher brain/peripheral ratio across all tested time points. Following intranasal delivery with small doses of BSA-LDHs-PHT, the latency of seizures in the pentylenetetrazole-induced mouse models was effectively improved. Collectively, the present study successfully designed and applied BSA-LDHs-PHT as a promising strategy for treating epileptic seizures with an enhanced therapeutic effect.

**Supplementary Information:**

The online version contains supplementary material available at 10.1186/s12951-024-02405-8.

## Introduction

Epilepsy is a common clinical syndrome defined by repetitive abnormal discharge of neurons in the brain and transient functional disturbance of the central nervous system [1, 2]. Epilepsy imposes significant burden and psychological pressure on patients and their family members with a high incidence ranging from 0.5 to 1% [3]. Currently, pharmacotherapy remains the primary approach for managing epilepsy. However, despite the vast array of anti-seizure medication (ASM) available, approximately 30% of patients experience “refractory epilepsy” [4]. This subset of patients is primarily characterized by suboptimal response to medication, including drug resistance and severe adverse drug reactions [5]. To enhance the risk reduction of seizures while minimizing the adverse effects associated with ASM, it is imperative to urgently address the crucial issue of improving the efficacy and safety of epilepsy medication in the current clinical scenario [6].

Phenytoin (PHT), the initial-generation ASM, has been subjected to clinical assessment for over fifty years, effectively inhibiting the propagation of abnormal neuronal discharges and managing focal and bilateral tonic-clonic seizures [7, 8]. Additionally, PHT can serve as a second-line therapeutic option for status epilepticus, offering a reduced likelihood of respiratory depression in comparison to benzodiazepines [9]. However, PHT is typically administered via oral or intravenous delivery in the clinic, so its limited solubility greatly reduces bioavailability [10]. Moreover, PHT acts as a substrate for both P-glycoprotein and multidrug resistance-associated proteins located on the blood-brain barrier (BBB), which reduces drug concentrations in the brain [11]. In addition, the metabolism of PHT is mediated by the cytochrome P450 enzyme system [12]. Due to the saturation of metabolic-inducing enzymes and extensive plasma protein binding, PHT exhibits a narrow therapeutic range. PHT has been partially replaced due to drug side effects, but the efficacy of the new generation of ASM has not exceeded that of PHT. Thus, the exploration of novel delivery methods is necessary to maximize the therapeutic ability of PHT in controlling epilepsy seizures and reducing the drug side effects.

Notably, intranasal (I.N.) delivery potentially offers a pathway to bypass the BBB and access the central nervous system via the olfactory nerves, presenting an alternative to conventional oral and intravenous drug delivery [13, 14]. By bypassing the BBB efflux transporter proteins [5], I.N. delivery can promote targeting efficiency in the brain and mitigate systemic side effects with lower dosages [15, 16]. At the same time, this non-invasive drug delivery method also provides a new application of phenytoin for pre-hospital emergency treatment of status epilepticus.

With the advancement of nanotechnology, various inorganic nanomaterials have been extensively applied in treating diverse diseases [17–19]. Among all, a two-dimensional inorganic nanomaterial, layered double hydroxide nanoparticles (LDH nanoparticles) have garnered significant attention as a promising delivering carrier [20, 21], with unique benefits including fine biocompatibility, mild toxicity, the ability to modify drug release, and ease of large-scale production [22]. We combined bovine serum albumin (BSA) with LDHs (BSA-LDHs) to endow the nanomaterial with colloidal stability [23]. Furthermore, the high positive charge and anion exchange properties exhibited by the surface of LDHs or BSA-LDHs render them suitable for efficiently loading and delivering charged therapeutic agents [24–26]. Notably, LDH nanoparticles have been extensively employed for transporting small compounds, proteins, and genetic material through the BBB to treat tumor and neurodegenerative conditions [27, 28], showing great potential as a desirable carrier for PHT.

**Fig. 1:**
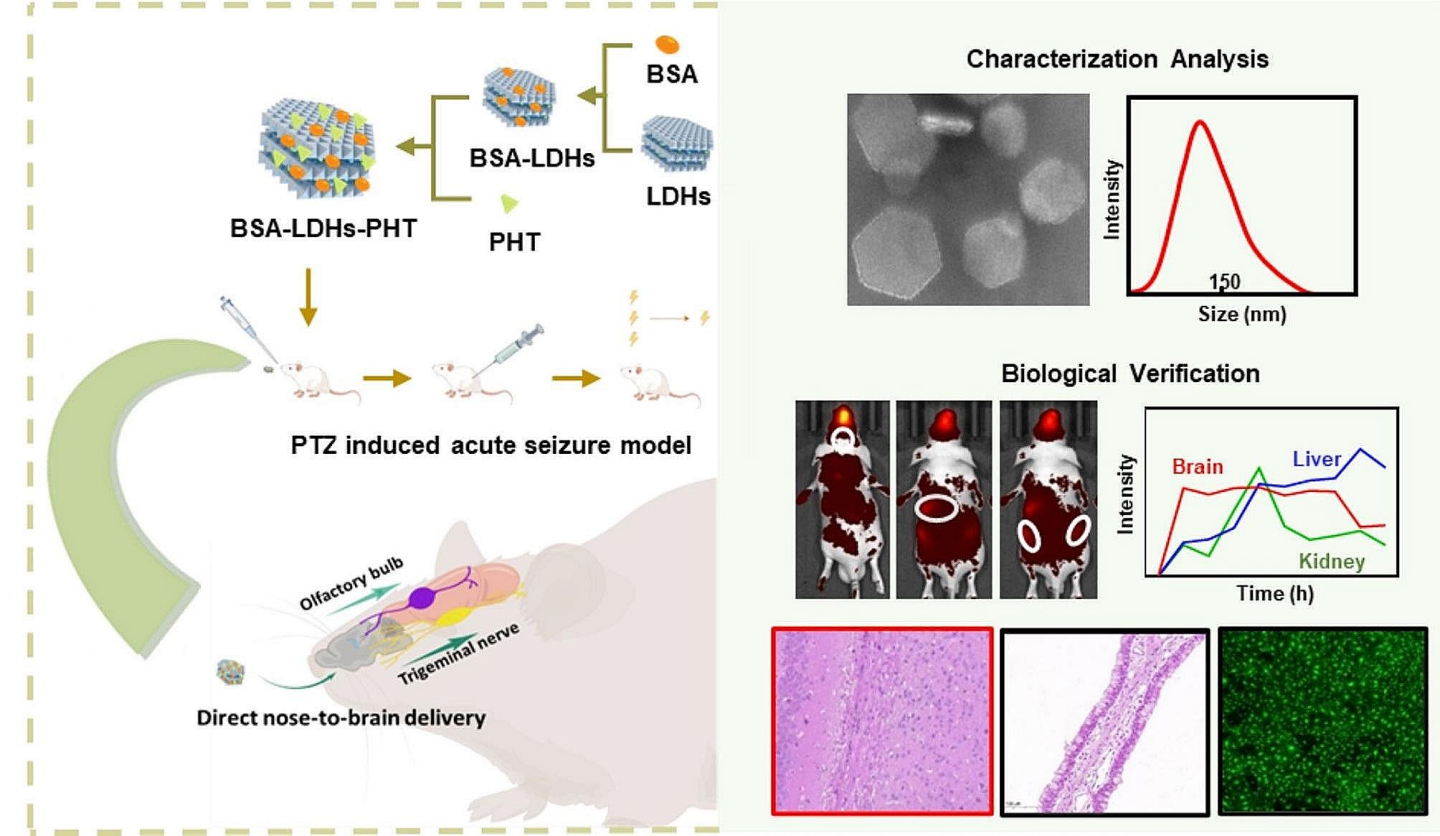
Schematic diagram of intranasal delivery of BSA-LDHs-PHT. The characterization analysis and biological verification demonstrated the efficacy and safety of epilepsy medication by this approach

In the current study, we developed BSA-LDHs loaded with phenytoin (BSA-LDHs-PHT) to facilitate the I.N. delivery of PHT bypassing the BBB to achieve seizure control (Fig. 1). According to in vivo fluorescence imaging assays, BSA-LDHs-PHT demonstrated a desirable capability for brain targeting. In addition, a PTZ-induced acute seizure model confirmed the excellent efficacy of I.N. delivery of BSA-LDHs-PHT. A 7-day repeated dosing study comprehensively demonstrated the safety profile of BSA-LDHs-PHT. The synergistic effect of nanoparticles combined with I.N. delivery presents novel prospects for enhancing the efficacy and ensuring the safe utilization of PHT, exhibiting great potential in managing epilepsy.

## Materials and methods

### Regents and materials

Phenytoin sodium was procured from Sigma Aldrich (USA). Beijing Chemicals (Beijing, China) provided MgCl_2_·6H_2_O, AlCl_3_·6H_2_O, and NaOH. Corning (USA) provided us with bovine serum albumin (BSA) and trypsin. Cell counting kit-8 (CCK-8) and antiseptic defibrinated sheep blood were sourced from Solarbio Life Sciences (China). PBS was obtained from Zhong Qiao Xin Zhou Biotechnology (China). BSA–ester Cyanine5.5 was procured from Xian ruixi Biological Technology Co., Ltd. (China). A Milli-Q water apparatus from Merck (Darmstadt, Germany) was utilized to produce ultra-pure water, employing a 0.22 μm filter.

### Cells and animals

Brain-derived endothelial cells.3 (Bend.3 cells, RRID: CVCL_0170) were purchased from Zhong Qiao Xin Zhou Biotechnology (China) and cultured in DMEM culture medium with 10% of FBS and 1% of penicillin/streptomycin. We obtained healthy male ICR mice or nude mice between 8 and 10 weeks old from certified local facilities (Zhejiang Vital River Laboratory Animal Technology Co., Ltd.). All mice were kept in a regulated environment, provided with sterile tap water ad libitum, and fed a standard rodent diet. The experimental animal ethics committee of Zhejiang University approved all experimental procedures, which were carried out in strict accordance with its guidelines.

### Preparation of BSA-LDHs-PHT

Firstly, to obtain LDHs, an alkaline solution containing NaOH was reacted with a salt solution comprising MgCl_2_ and AlCl_3_, according to previous works [21]. After 15 min of intense stirring, the resulting slurry was harvested, washed, and then exposed to hydrothermal processing at a temperature of 100℃ for 16 h. BSA was dissolved in deionized water. To ensure BSA-coated LDHs’ stability, pristine LDHs were gradually added dropwise into an equal volume of BSA under continuous magnetic stirring for 5 min. Excess BSA was removed by centrifuging the resulting BSA-LDHs (Additional file 1: Fig S1).

Secondly, deionized water was used to dissolve phenytoin sodium at a concentration of 4 mg/mL. This solution was then utilized to resuspend the BSA-LDHs, resulting in the formation of phenytoin-loaded BSA-LDHs (BSA-LDHs-PHT). The BSA-LDHs-PHT solutions were evenly distributed through ultrasonication with a probe sonicator.

To find the optimal synthesis mass ratio between phenytoin sodium solution and BSA-LDHs, different mass ratios of PHT/BSA-LDHs, ranging from 1:2 to 1:6, were investigated. The mixtures of different mass ratios were subjected to centrifugation at 20,000 g for 20 min, and the resulting supernatants were collected. The NanoDrop (Thermo Scientific) was utilized to measure the absorbance of PHT in the supernatant at a wavelength of 191 nm. The amount of PHT was calculated using a linear standard curve. The rate of drug adsorption was determined by utilizing the equation provided below:

$$\% \,{\rm{Drug}}\,{\rm{adsorption}}\,{\rm{rate}}\,{\rm{ = }}\,\left( {{{{\rm{Amount}}\,{\rm{of}}\,{\rm{drug}}\,{\rm{entrapped}}} \over {{\rm{Amount}}\,{\rm{of}}\,{\rm{drug}}\,{\rm{taken}}\,{\rm{initially}}}}} \right)\, \times \,{\rm{100}}\%$$

### Nanoparticles characterization of BSA-LDHs-PHT

#### TEM images of BSA-LDHs and BSA-LDHs-PHT

The BSA-LDHs and BSA-LDHs-PHT samples were subjected to negative staining with phosphotungstic acid and imaged using an HT7800 Ruli TEM with an acceleration voltage of 80 kV.

### Drug loading efficiency of BSA-LDHs-PHT

PHT was detected using the NanoDrop (Thermo Scientific) at 191 nm. The PHT concentration was determined by calculating it using a linear standard curve. The drug loading efficiency was calculated using the following equation:

$$\% \,{\rm{Drug}}\,{\rm{loading}}\,{\rm{Efficiency}}\,{\rm{ = }}{\mkern 1mu} {{\left( {{\rm{Dru}}{{\rm{g}}_{{\rm{Total}}}}{\rm{ - Dru}}{{\rm{g}}_{{\rm{filtrate}}}}} \right)} \over {{\rm{Dru}}{{\rm{g}}_{{\rm{Total}}}}}}\, \times \,{\rm{100}}$$

### Particle size, polydispersity index (PDI), and Zeta potential

The Zetasizer Nano ZS apparatus (Malvern, UK) automatically measured the mean particle size, polydispersity index (PDI), and zeta potential of each sample in triplicate. Prior to measuring, the nanoparticle suspension was thinned by a factor of 50 using de-ionized water. Scattered light was collected at a 90° angle for a duration of 2 min during the measurements conducted at a temperature of 25℃.

### Stability analysis of BSA-LDHs-PHT

The stability of LDH, BSA-LDHs, and BSA-LDHs-PHT in serum was assessed by dispersing the nanoparticles in either water or FBS (1 mg/mL) for 24 h. The size of the nanoparticles was measured using DLS.

The BSA-LDHs-PHT samples were stored at a temperature of 4$\pm$2℃. The size and zeta potential were assessed at predetermined time intervals of 1, 7, 14, and 21 days [29].

### XRD pattern of BSA-LDHs-PHT

A D8 ADVANCE X-ray diffractometer was used to record the X-ray diffraction (XRD) patterns, using wide-angle diffraction at 40 kV and 40 mA.

### FTIR analyses of BSA-LDHs-PHT

Fourier transform infrared (FTIR) analyses of BSA-LDHs-PHT, BSA-LDHs, and PHT were performed using a Fourier transform infrared Spectrophotometer (INVENIO, Germany) to investigate potential interactions between the drugs and excipients.

### Drug release study

To study release kinetics, an appropriate amount of BSA-LDHs-PHT (equivalent to 20 µg PHT) was suspended in 1 mL of deionized water and incubated at 4℃. The suspension was centrifuged at 20,000 rpm for 20 min at specific time intervals (0.5, 1, 2, 4, 6, 8, 12, 24, 48, 72 h). Afterward, 0.2 mL of the supernatant was gathered and exchanged with deionized water. The drug concentration in the collected samples was analyzed using the NanoDrop (Thermo Scientific) at a wavelength of λ = 191 nm [30, 31].

### In vitro cytotoxicity studies

#### Calcein-AM/PI fluorescence staining

The staining method of Calcein-AM/PI was employed to label living cells and cells undergoing necrosis. Bend.3 cells were grown in DMEM medium with a cell density of 20,000 cells. 24 h after seeding, the cells were treated with DMEM medium containing 200 µg/mL of phenytoin sodium solutions, BSA-LDHs solutions or BSA-LDHs-PHT solutions respectively for 24 or 48 h. The control group consisted of cells treated with pure DMEM medium. After 24 or 48 h, we removed the 24-well plates and washed them twice with PBS. Then the plates were added 250 µL Calcein-AM/PI per well and incubated at 37℃ for 30 min. Finally, we used an inverted fluorescence microscope to observe the cells.

### CCK-8 assay

Bend.3 cells were cultured in the same way as above at a density of 8000 cells/well. Following a 24-hour seeding period, the cells underwent treatment with growth medium that included varying concentrations (10, 50, 100, or 200 µg/mL) of phenytoin sodium solutions, BSA-LDHs solutions, or BSA-LDHs-PHT solutions for either 48 h. Cytotoxicity was evaluated using the CCK-8 assay by measuring absorbance at 450 nm on a multifunction measuring instrument (Biotek). Cell viability was performed by utilizing the subsequent formula:

$$\% \,{\rm{Cell}}\,{\rm{viability}}\,{\rm{ = }}{\mkern 1mu} \,\left( {{{{\rm{Absorbance}}\,{\rm{in}}\,{\rm{the}}\,{\rm{treatment}}\,{\rm{well}}} \over {{\rm{Absorbance}}\,{\rm{in}}\,{\rm{the}}\,{\rm{control}}\,{\rm{well}}}}} \right) \times {\rm{100}}\% {\rm{ }}$$

### Hemolysis assay

Antiseptic defibrinated sheep blood was diluted in PBS (1 mL blood in 10 mL PBS). The concentrated red blood cells (RBCs) were separated by spinning at a speed of 20,000 revolutions per minute for a duration of 20 min and then rinsed more than five times with sterile isotonic PBS until the absence of any red hue in the liquid above. Then, 200 µL of RBCs were diluted into 4 mL of PBS. The diluted RBC suspension (0.2 mL) was then mixed with LDHs, BSA-LDHs, or BSA-LDHs-PHT solutions (0.8 mL) at various concentrations ranging from 10 to 800 µg/mL. Instead of using LDHs solution, PBS and water (0.8 mL) were employed as the positive and negative control, respectively. After gently shaking the mixtures, they were incubated at room temperature for 2 h. Then, they were centrifuged at a speed of 1600 rpm for 5 min. A multifunction measuring instrument (Biotek) was used to measure the absorbance of the supernatant at 560 nm. The calculation of the percentage of red blood cell hemolysis was performed using the formula [28]:

$${\rm{Hemolysis}}\,{\rm{percentage}}{\mkern 1mu} {\rm{ = }}{\mkern 1mu} {{({\rm{Ab}}{{\rm{s}}_{{\rm{sample}}}}{\rm{ - Ab}}{{\rm{s}}_{{\rm{negativecontrol}}}})} \over {\left( {{\rm{Ab}}{{\rm{s}}_{{\rm{positivecontrol}}}}{\rm{ - Ab}}{{\rm{s}}_{{\rm{negativecontrol}}}}} \right)}} \times {\rm{100}}\%$$

### In vivo fluorescence imaging

To investigate the nose-to-brain delivery capacity of the BSA-LDHs, we utilized the fluorescent dye Cyanine5.5 (Cy5.5) as a tracer for live imaging of the entire body in vivo [32]. Prior to use, LDH nanoparticles were linked with BSA-Cy5.5, and any surplus dye molecules were eliminated through centrifugation. Male mice were randomly divided into two treatment groups (*n* = 6): (1) control group (I.N. administration of BSA-Cy5.5 solutions), (2) LDH nanoparticles group (I.N. administration of BSA-LDHs-Cy5.5 solutions). At predetermined time points following I.N. administration (15 min, 30 min, 1 h, 2 h, 4 h, 8 h, 12 h, 24 h), the mice were anesthetized and placed in an IVIS Spectrum animal imager to capture the fluorescence images. Regions of interest including the brain, liver, kidneys, and the entire mouse body excluding the brain were delineated on these images to quantify fluorescence intensity at each time point.

After 15 min of intranasal administration, the mice received a saline infusion, and the respective brains, hearts, livers, spleens, lungs, and kidneys were subsequently prepared. The IVIS Spectrum animal imager was utilized to perform fluorescence imaging on separated organs.

### In vivo pharmacokinetic study of BSA-LDHs-PHT

Intranasal administration of BSA-LDHs-PHT solutions in healthy female ICR mice (20–30 g). At predetermined time points following intranasal administration (30 min, 1 h, 2 h, 4 h, 8 h), the mice were anesthetized and collected their blood samples and brain tissues (*n* = 3 for each time point).

Acetonitrile and methanol were used as solvents for extracting phenytoin from plasma and brain homogenates. Aliquots of 5 µL of each processed sample after filtration through 0.22 μm syringe filter were injected into the chromatographic system for LC-MS/MS (AB QTRAP 5500) analysis using a C18 column (Waters ACQUITY UPLC BEH C18 1.7 μm, 2.1*50 mm). The flow rate was kept constant at 0.3 mL/min, and the temperature was maintained at 40 °C (Mobile Phase A: Contained 0.1% FA and 5mM NH4Ac in water, Mobile Phase B: Contained 0.1% FA in MeOH). The MS condition is an electrospray ion (ESI) source. Positive and negative ion multi-reaction ion monitoring (MRM) was used for quantitative analysis. All concentrations were calculated from a standard curve of PHT obtained from spiked tissue samples.

### PTZ-induced acute seizure model

In this investigation, a total of 40 ICR mice were randomly divided into four groups (*n* = 10) and subjected to various administration schedules. (1) I.N. delivery of normal saline (50 µL per mouse); (2) oral delivery via gastric irrigation of PHT solution (1 mg/mL, 200uL per mouse); (3) I.N. delivery of PHT solution (4 mg/mL, 50 µL per mouse); (4) I.N. delivery of BSA-LDHs-PHT solution (4 mg/mL, 50 µL per mouse). For I.N. administration, 25 µL of the drug solution was administered into each nostril using a 100µL pipette gun, while the animal was positioned in a supine position. We dropped the medication onto the nostrils of mice and let them inhale naturally.

After 5 or 30 min of administration, all groups received an intraperitoneal injection of PTZ (100 mg/kg) and were assessed for the intensity of seizures within the subsequent 30-minute timeframe. The severity of seizures was evaluated using the Racine scale [33]: (1) immobility and facial movement, (2) nodding of the head, (3) unilateral forelimb clonus, (4) bilateral forelimb clonus with rearing, (5) jumping and falling, (6) tonic-clonic convulsions and extension of the hindlimbs. Furthermore, we recorded the typical electroencephalogram (EEG) of the PTZ-induced acute seizure model to show the severity of epileptic discharge at different stages. The study groups’ preparations and routes of administration details are displayed in additional file 1: Table S1. We measured the pesticide effect by recording the latency duration of different seizure stages in mice after administration.

### Toxicity assessment of BSA-LDHs-PHT

The toxicities of BSA-LDHs-PHT on the nasal mucosa and other major organs (brain, heart, liver, and kidneys) were assessed through histopathology using hematoxylin and eosin (H&E) staining. At the same time, we evaluated the toxicity of drugs to specific systems by testing blood routine and liver and kidney function. For this purpose, eight adult ICR male mice were stochastically divided into two groups (*n* = 4) and intranasally administered with 50 µL per mouse of either normal saline (negative control) or BSA-LDHs-PHT solutions once daily for seven consecutive days. Animal weight was monitored daily before administration. 24 h after the final administration, mice were anesthetized and mice’s blood was collected for blood routine and liver and kidney function testing. Then mice were subjected to a cardio-perfusion technique using 4% polyformaldehyde following a rinse with normal saline for 20 min to prevent tissue autolysis post-mortem. To prevent harm, the organs and nasal passages were carefully removed and preserved in a solution of 4% polyformaldehyde for 72 h. Then the tissues underwent dehydration in ethanol and were embedded in paraffin wax. The paraffin blocks were cut horizontally into tissue sections that were 5 μm thick using a micrometer. These sections were then stained with H&E and examined under an optical microscope [34].

### Statistical analysis

Statistical analysis was performed on the data using either the t-test or one-way ANOVA. A statistical significance was attributed to a significance level lower than 0.05, denoted as *p* < 0.05.

## Results and discussion

### Preparation and characterization of BSA-LDHs-PHT

**Fig. 2:**
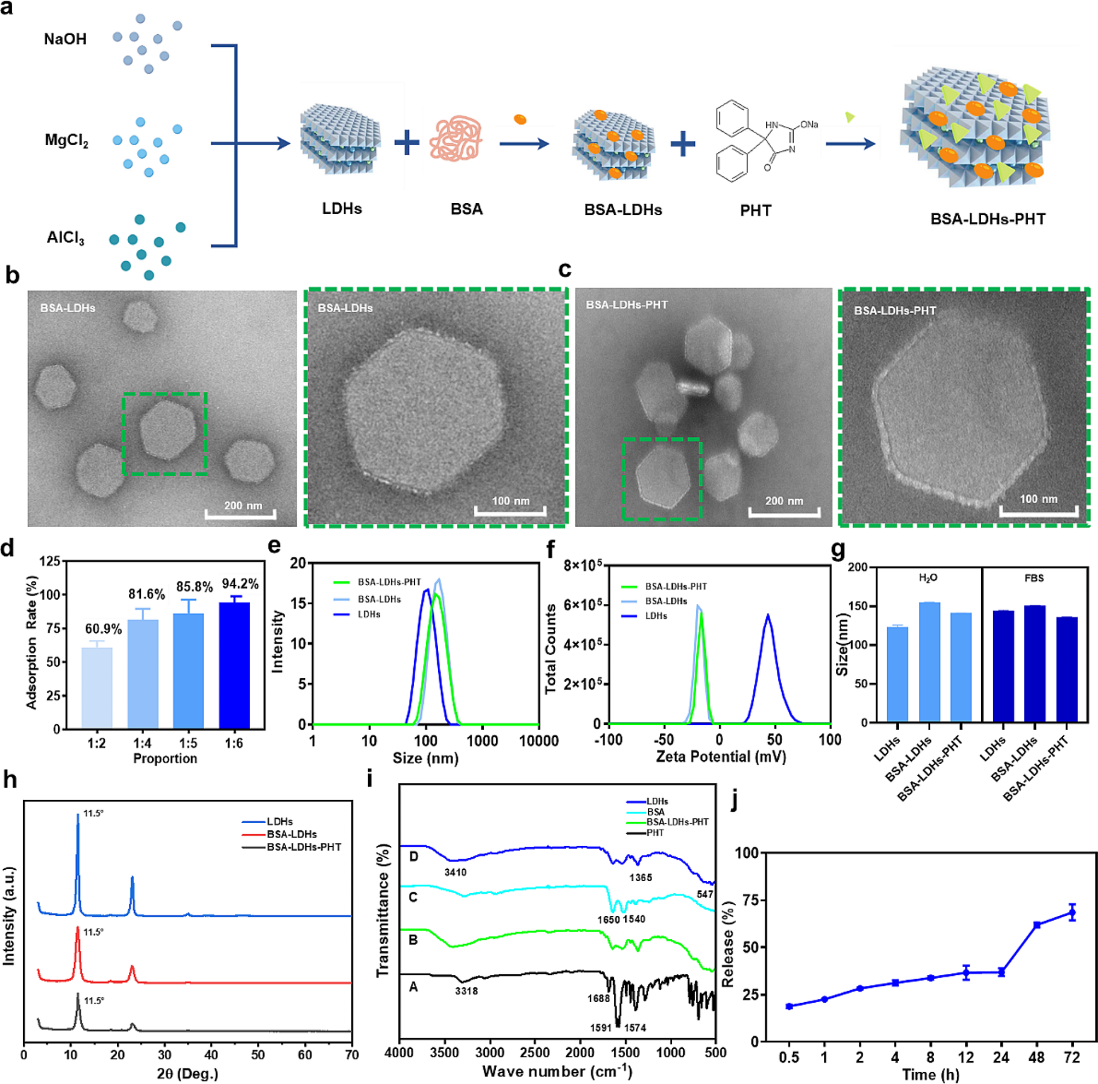
Characterization of BSA-LDHs-PHT. **a** Schematic diagram of the composition of BSA-LDHs-PHT. **b, c** TEM micrographs of BSA-LDHs and BSA-LDHs-PHT. **d** The drug adsorption rate increases as the mass ratio of PHT/BSA-LDHs increases from 1:2 to 1:6. **e, f** The profile analyzed by DLS shows the average size and zeta potential of LDHs, BSA-LDHs, and BSA-LDHs-PHT. **g** The colloidal stability of LDHs, BSA-LDHs, and BSA-LDHs-PHT in water and FBS for 24 h. **h** XRD patterns of LDHs, BSA-LDHs, and BSA-LDHs-PHT. **i** FTIR analysis of BSA-LDHs-PHT. **j** PHT was progressively released from the nanoparticles, reaching 36.91 ± 2.07% of the initially encapsulated drug within 24 h. The data presented were the mean ± SD (*n* = 3)

As shown in Fig. 2a, we developed PHT loaded layered double hydroxide nanoparticles (BSA-LDHs-PHT) via a coprecipitation-hydrothermal method. To achieve homogenous LDH suspensions, we employed BSA as a stabilizing agent for LDHs (BSA-LDHs) [23]. The TEM images (Fig. 2b, c) revealed that the BSA-LDHs and BSA-LDHs-PHT exhibited a characteristic hexagonal lamellar structure, with diameters around 100 ∼ 200 nm and no apparent aggregation. Notably, no discernible presence of PHT crystals was observed in the background. Figure 2d showed that the adsorption rate of PHT increased significantly with the mass of BSA-LDHs. Considering the effects of adsorption rate and material dosage, a mass ratio of 1:5 (PHT to BSA-LDHs) was selected as the standard for synthesizing BSA-LDHs-PHT, ensuring optimal adsorption efficiency while minimizing material consumption. The average percentage drug loading efficiency of BSA-LDHs-PHT is 34.86%.

As depicted in Fig. 2e, the average particle size of uniformly dispersed BSA-LDHs-PHT in water was determined to be 146.5 ± 3.2 nm, with a PDI value of 0.24, indicating a homogeneous particle size distribution. The small nanoparticle size facilitates the diffusion of BSA-LDHs-PHT through nasal mucus, enabling direct interaction with the lower epithelium for brain delivery [35]. By loading varying quantities of anionic medications, the zeta potential of LDH-drug hybrid nanoparticles can be controlled [43]. Due to the presence of negatively charged BSA, anionic zeta potential (-16.6 ± 0.1 mv) was observed for the BSA-LDHs-PHT solution (Fig. 2f). With a negative potential, BSA-LDHs-PHT will not be attracted by the mucin, so it does not stay in the nasal cavity for too long and possess greater freedom of movement. By adopting this approach, PHT could readily reach the brain. When BSA-LDHs-PHT enter the brain, they directly release phenytoin in the cerebrospinal fluid, causing it to bind to Na^+^ channels on the surface of neurons and control epileptic seizures [36]. By carrying the negative charges, nanoparticles are not easily absorbed by cells, allowing phenytoin to be directly released in cerebrospinal fluid and better exert its effects on the surface of the neuronal plasma membrane. Colloidal stability analysis showed that BSA-LDHs and BSA-LDHs-PHT were stably dispersed in water and FBS (Fig. 2g). At a storage temperature of 4℃, no significant change in the size and zeta potential of nanoparticles was observed within 21 days of the freshly prepared nanoparticles (Additional file 1: Fig S2).

LDHs, BSA-LDHs, and BSA-LDHs-PHT exhibit the typical layered features from XRD patterns (Fig. 2h) and demonstrate good crystallinity, indicating the loading of PHT did not affect LDHs structure. As depicted in Fig. 2i, FTIR spectroscopy illustrates the absorption peaks of PHT, LDHs, BSA, and BSA-LDHs-PHT. Specifically, the spectra of PHT exhibited the stretching vibration of the carbonyl group at 1688 cm^− 1^, accompanied by a shoulder vibration at 1674 cm^− 1^ [37]. The characteristic bands for BSA were observed at 1650 cm^− 1^ (C = O stretching vibration) and 1540 cm^− 1^ (C-N stretching vibration) respectively [38]. In the FTIR spectra of BSA-LDHs-PHT, ratio peaks originating from both PHT and BSA can be identified, indicating successful adsorption onto the LDHs surface [39].

The releasing behavior of PHT from BSA-LDHs-PHT was further determined (Fig. 2j). The release rate of PHT reached about 36.91 ± 2.07% at 24 h and 68.58 ± 4.25% at 72 h. BSA-LDHs exhibited a biphasic release behavior for PHT: an initial rapid release followed by a subsequent slow steady release. This can be attributed to LDHs releasing anions in cerebrospinal fluid with a pH of 7.4. The slight initial release burst may result from the easy exchange of surface-bound PHT molecules on nanoparticles, while subsequent diffusion occurs within the core and eventually into solution over 24–72 h [22]. This finding has implications for predicting the behavior of I.N. delivered BSA-LDHs-PHT.

### Biocompatibility of BSA-LDHs-PHT

**Fig. 3:**
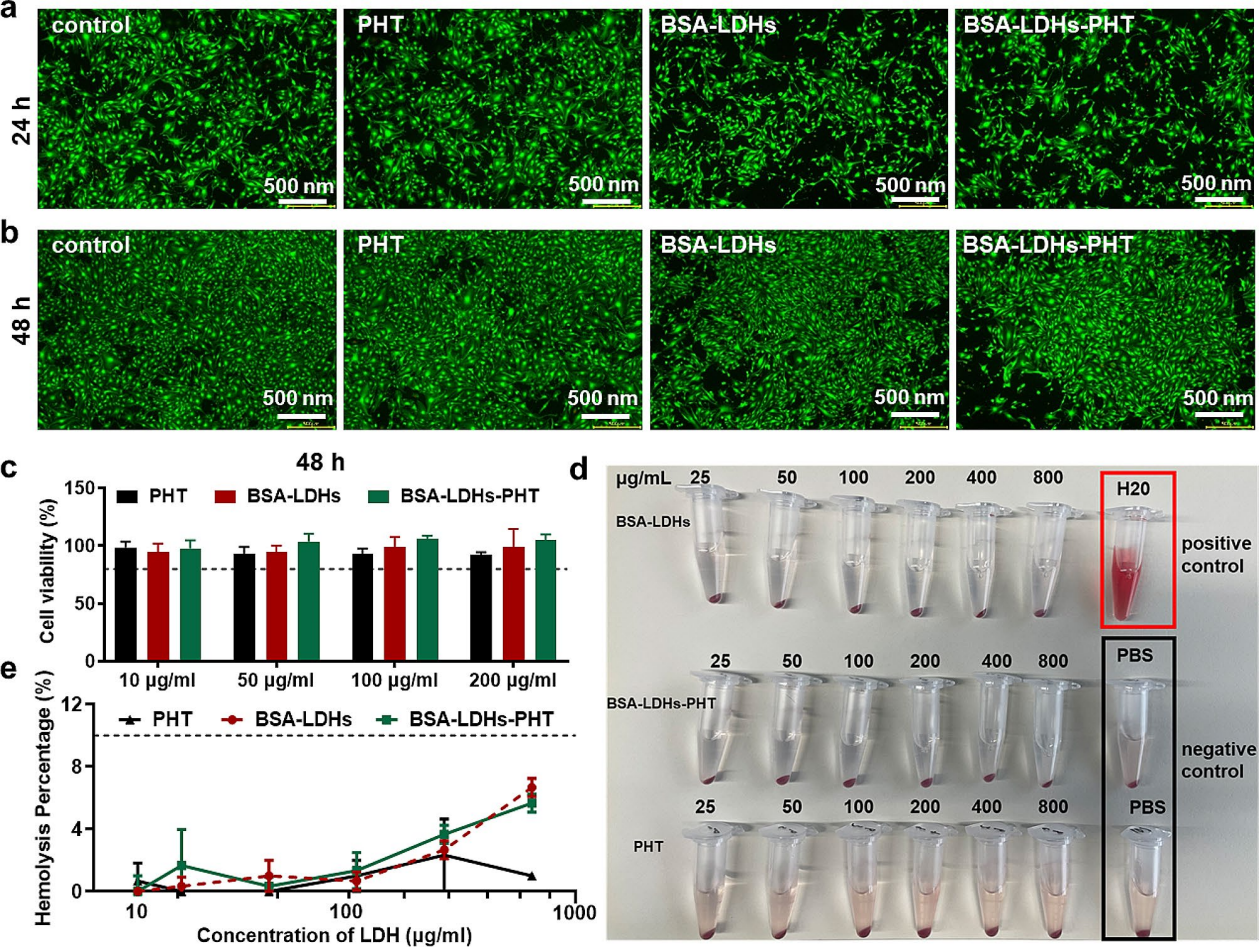
Calcein-AM/PI fluorescence staining, CCK-8 assay, and hemolysis test of LDHs-BSA-PHT. **a, b** Calcein-AM/PI fluorescence staining after 24 **(a)** and 48 **(b)** h, most of the cells in the experimental groups (PHT, BSA-LDHs, BSA-LDHs-PHT) showed green, which is similar to the blank control group. **c** Cell viability of Bend.3 cells were treated with PHT, BSA-LDHs, and BSA-LDHs-PHT at 0-200 µg/mL. No significant distinction was observed between the experimental and control groups (*p* > 0.05). **d, e** Photographs **(d)** and hemolysis assay **(e)** of RBCs treated with nanoparticles at 0 − 800 µg/mL. The experiments were conducted in pairs and replicated thrice. The experimental and negative control groups did not show any significant difference (*p* > 0.05). The data provided was the mean ± SD (*n* = 3). * *p* < 0.05; ** *p* < 0.01; *** *p* < 0.001

As shown in Fig. 3a, b, after staining with Calcein-AM/PI, most cells in the experimental groups (PHT, BSA-LDHs, BSA-LDHs-PHT solution) exited green fluorescence after 24 and 48 h, similar to the blank control group, intuitively demonstrating a high cell survival rate. In the CCK-8 assay, following a culture period of 48 h, all treatment groups (LDHs concentrations ranging from 10 to 200 µg/mL) showed over 90% cell viability for Bend.3 cells exposed to LDHs preparations (Fig. 3c). Our results indicated that BSA-LDHs-PHT exhibited excellent biocompatibility with Bend.3 cells [21]. Compared to commonly used polymer carriers such as polyethyleneimine, BSA-LDHs are more than 10 times less toxic [40].

As shown in Fig. 3d, the maximum hemoglobin release from damaged cells occurred from the positive control group (H_2_O). Other groups (PHT, BSA-LDHs, BSA-LDHs-PHT solution) exhibited nearly colorless supernatants at all concentrations. As depicted in Fig. 3e, even at a concentration of 800 µg/mL, the hemolysis rate of nanomaterials remained below 10%. The above results align with previous findings [28, 41], indicating favorable blood biocompatibility of our nanoparticles.

### Brain-targeted delivery mediated by BSA-LDHs-PHT

**Fig. 4:**
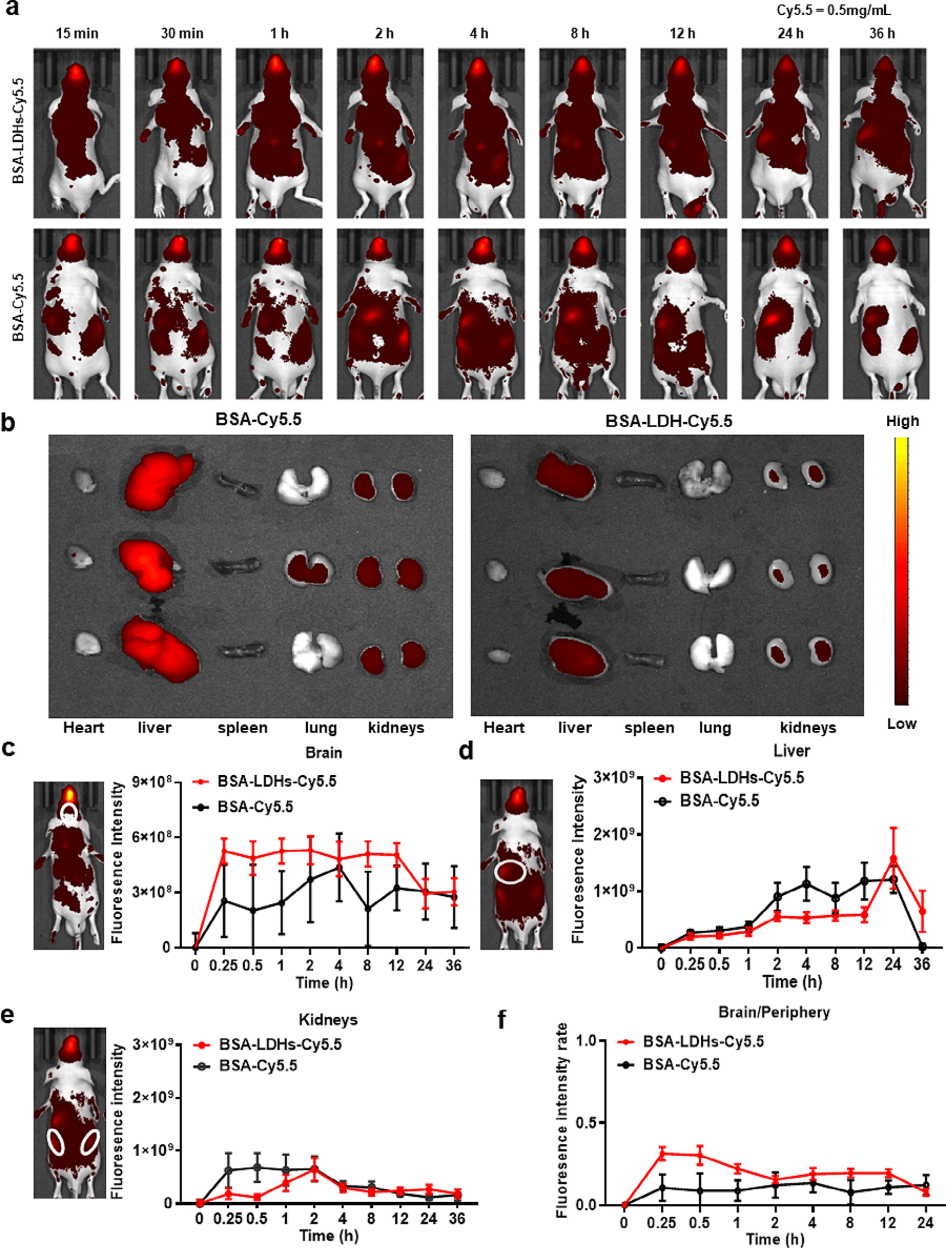
The fluorescence intensity distribution in the brain and major organs following BSA-LDHs-Cy5.5 I.N. administration. **a** Fluorescence imaging at specific time points after I.N. delivery (*n* = 6). **b** In vitro fluorescence images of mice organs (hearts, livers, spleens, lungs, and kidneys) after 15 min of administration (*n* = 3). **c, d, e** Schematic diagrams of the brain **(c)**, liver **(d)**, and kidneys **(e)** frame selection for fluorescence in vivo imaging and the mean brain, liver, and kidneys fluorescence intensity following I.N. delivery of BAS-LDHs-Cy5.5 and BSA-Cy5.5 (*n* = 6). **f** The fluorescence intensity ratio (brain/periphery) associated with BAS-LDHs-Cy5.5 was higher than that measured for BSA–Cy5.5 from 15 min to 24 h (*n* = 6). Data are represented as fluorescence intensity, expressed as mean ± sem. * *p* < 0.05; ** *p* < 0.01; *** *p* < 0.001

Nanoparticles in vivo distribution experiments show a good brain targeting performance of BSA-LDHs. Figure 4a showed the fluorescence images of systemic drug distribution in two groups of mice at different times after I.N. administration. We can preliminarily see that compared to BSA-Cy5.5, BSA-LDHs-Cy5.5 were more concentrated in the brain rather than the periphery. We took in vitro fluorescence images of mice brains (Additional file 1: Fig S3) and peripheral organs (hearts, livers, spleens, lungs, and kidneys) (Fig. 4b) after 15 min of administration. In Fig. 4b, we can see that in the control group, a large amount of BSA-Cy5.5 accumulates in peripheral organs such as the liver and kidneys, while in the experimental group, the aggregation is much less, further indicating that more BSA-LDH-Cy5.5 accumulates in the brain. This conclusion has been confirmed in in vivo brain imaging at different time points. Unfortunately, due to the fluorescence quenching effect [42], we did not find the corresponding differences between the two groups of in vitro brain imaging (Additional file 1: Fig S3).

Figure 4c shows the fluorescence intensity of in vivo brain imaging at different time points. Compared to BSA-Cy5.5, BSA-LDHs-Cy5.5 enters the brain faster and at higher concentrations, demonstrating superior brain targeting properties. After 15 min of intranasal administration, the fluorescence intensity of BSA-LDHs-Cy5.5 (5.25 × 10^8^) was twice higher than that of BSA-Cy5.5 (2.54 × 10^8^). This can be attributed to the inherent advantages of LDHs as an in vivo drug carrier, including their small molecular weight, compact size, excellent transmembrane capability, and facile traversal of tight junctions present in the nasal epithelium [43]. Additionally, the hydroxide layers of LDH nanoparticles could protect loading drugs from enzymatic degradation at both the nasal mucosa and blood-brain barrier, leading to improved drug release efficiency with fewer off-target effects [22].

We further conducted pharmacokinetic experiments on phenytoin. Additional file 1: Fig. S4 a shows the brain concentration-time profiles of phenytoin after intranasal administration of BSA-LDHs-PHT. After half an hour of intranasal administration, we were able to detect a certain concentration of phenytoin in brain tissues. The highest whole-brain drug concentration for phenytoin was 404.3 ng/mL at 4 h. After 8 h, phenytoin could still maintain a high drug concentration in the brain. The above results further intuitively indicate that nanoparticles rapidly enter the brain and continuously release phenytoin for a long time, which is consistent with the results of mice fluorescence imaging.

PHT was previously a widely utilized ASM, but its usage has gradually declined due to limitations imposed by its adverse effects [44]. Individuals with hepatic or renal insufficiency are more susceptible to PHT adverse effects as it is metabolized by cytochrome P450 enzymes in the liver and excreted through the kidneys [45]. With increased PHT dosage, plasma drug concentrations rise disproportionately, potentially leading to toxicity within the body. If we can improve drug delivery efficiency and reduce drug peripheral distribution, it will reduce the systemic side effects of PHT. Therefore, it is crucial to verify the ratio of BSA-LDHs-Cy5.5 in organs beyond brain sites after I.N. administration. As depicted in Fig. 4d, e, fluorescence intensities of BSA-LDHs-Cy5.5 were lower in the liver and kidney compared to those observed in the BSA-Cy5.5 group at various time points after I.N. injection. After 4 h of intranasal administration, the fluorescence intensity of BSA-LDHs-Cy5.5 (2.13 × 10^8^) in the liver was half that of BSA-Cy5.5 (5.34 × 10^9^). In the kidneys, 0.5 h after administration, the fluorescence intensity of the nanomaterial group was only one-fifth that of the control group. As shown in Fig. 4f, the fluorescence intensity ratio (brain/periphery) of LDHs-BSA-Cy5.5 was higher than BSA-Cy5.5 from 15 min to 12 h, indicating that nanomaterials are more concentrated in the brain than in peripheral tissues. We synchronously detected the concentration of phenytoin in the plasma after intranasal delivery of BSA-LDHs-PHT. As shown in additional file 1: Fig. S4 b, the concentration of phenytoin in the plasma remains at a relatively low level (C_max_ = 1.84 µg/mL). It is lower than the plasma concentration of phenytoin (5–20 µg/mL) detected in another study which is about phenytoin loaded electro-responsive hydrogel nanoparticles [46]. This observation is attributed to the exceptional brain-targeting ability of nanomaterials. Higher drug delivery efficiency means that effective treatment can be achieved with fewer doses. Combined with the nasal administration strategy, the drug could bypass the hepatic first-pass effect and direct entry into the brain at a lower dose level. This approach would effectively mitigate both the concentration and potential toxicity associated with PHT, offering a promising avenue for its safe utilization.

### Epileptic seizure control of BSA-LDHs-PHT I.N. delivery

**Fig. 5:**
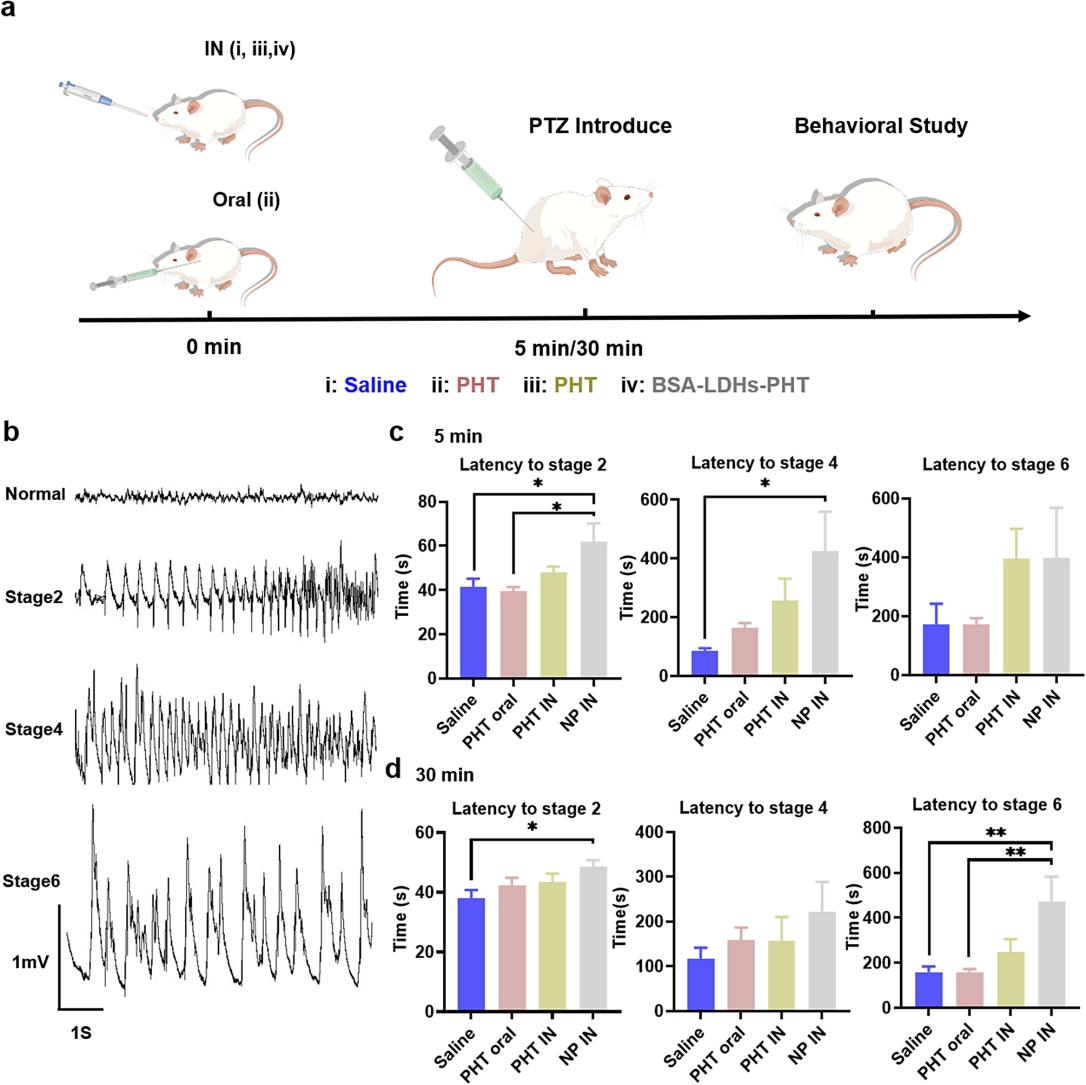
Epileptic seizure control of BSA-LDHs-PHT I.N. delivery. **a** Schematic diagram of PTZ-induced acute seizures after 5 and 30 min of administration. **b** The representative EEGs of normal mice and epileptic mice at stage 2, 4, and 6. **c** Statistics of the latency of I.N., PHT oral, PHT I.N., and BSA-LDHs-PHT I.N. after 5 min of administration. **d** Statistics of the latency of I.N., PHT oral, PHT I.N., and BSA-LDHs-PHT I.N. after 30 min of administration. Data expressed as mean ± sem (*n* = 10). * *p* < 0.05; ** *p* < 0.01; *** *p* < 0.001. NP nanoparticle, IN intranasal

PHT exhibits favorable therapeutic efficacy for epilepsy, however, its effective concentration range is narrow (10–20 µg/mL), and exceeding this range may lead to toxic reactions. Therefore, we investigated the potential of reducing the dosage of PHT through a combination of I.N. delivery and nanomaterial technology. Previous research has shown that a conventional PHT dose of 20–50 mg/kg reduces seizure severity in the PTZ-induced mouse model [46]. In this study, we aimed to evaluate the efficacy of I.N. delivery of BSA-LDHs-PHT by reducing the PHT dosage to 4 mg/kg and investigated different modes of administration (oral and nasal) as well as drug formulations on seizures in the PTZ-induced mouse model (Fig. 5a). The PTZ-induced acute seizure model is widely employed in animal studies. We recorded the representative EEGs (Fig. 5b) of normal mice and epileptic mice at stage 2, 4, and 6. The mice were divided into four groups, and the specific administration methods and doses are presented in additional file 1: Table S1. The latencies of different convulsive phases (2, 4, and 6) were recorded in each group of mice following drug administration.

After a five-minute administration period, the latency of stages 2 and 4 exhibited significant prolongation in the BSA-LDHs-PHT I.N. delivery group compared to the saline group (Fig. 5c, *p *$\text{<}$ 0.05). Furthermore, I.N. delivery demonstrated significantly superior prolongation of phase 2 latency when compared to oral delivery (Fig. 5c, *p *$\text{<}$ 0.05). These results indicate that BSA-LDHs-PHT rapidly enters the brain and exerts anti-epileptic effects after being delivered to the nasal cavity of mice. Many previous studies showed that I.N. administration has a good delivery efficiency in delivering therapeutic agents to the brain and positively affects brain-related diseases [47, 48]. We know that the BBB composed of endothelial cells that form central nervous system blood vessels, possesses a stronger selective filtration function than other systemic blood vessels within our body. While it prevents harmful substances from entering the central nervous system on one hand, it also restricts drug entry from the bloodstream into the interstitial space via systemic pathways. The nose-brain pathway represents a novel route for targeted drug delivery to the central nervous system. Because the nose is the only area of direct contact between the brain and the external condition. When the drug reaches the nasal mucosa and contacts the olfactory nerve, it can directly enter the central nervous system through the holes of the lamina cribrosa and bypass the BBB [14]. In Fig. 5b, BSA-LDHs-PHT exhibited therapeutic effects within 5 min of I.N. administration. Due to its noninvasive nature, I.N. delivery offers a promising approach for utilizing PHT in prehospital emergency treatment of persistent status epilepticus. The lower effect after oral and nasal use of PHT solution compared with nasal use of BSA-LDHs-PHT could be attributed to the limited water solubility of PHT. PHT likely created a fully saturated solution at the site of absorption [49]. On the contrary, packaging PHT in BSA-LDHs with small size, extensive surface area, and controlled drug release could prevent the formation of PHT precipitates and improve the efficacy of PHT [50].

After 30 min of administration, BSA-LDHs-PHT still showed stable effects. In stage 6, the incubation period of BSA-LDHs-PHT was extended more than twice compared to the saline and oral PHT group (Fig. 5d, *p *$\text{<}\text{ }$0.01). This was attributed to the unique characteristics of BSA-LDHs. In the previous in vitro experiments, we simulated the release of phenytoin from BSA-LDHs. The initial burst release allows for a rapid onset of action of BSA-LDHs-PHT, and the subsequent slow-release effect maintains in vivo drug concentrations at appropriate levels and exerts long-term effects. In the fluorescence imaging experiment mentioned in the previous section, our nanomaterials could remain in the brain for up to 24 h. The combination of nanomaterials and I.N. delivery has been a research hotspot in recent years. In a 2021 article about IN delivery of phenytoin-loaded nano lipid carriers, in vivo pharmacokinetic studies confirmed that phenytoin-loaded nano lipid carriers can reach the brain within 5 min upon I.N. administration [16]. However, the concentration of phenytoin in the brain rapidly decreases within 15 min in this research, which limits the application of this material in the treatment of chronic epilepsy. Our nanomaterials are expected to play a certain role in both acute epileptic seizure control and chronic epilepsy treatment. Unfortunately, we did not try to observe the efficacy after a longer administration time in this article, which would become one of the experimental directions worth studying in the future.

### Toxicity assessment of BSA-LDHs-PHT

**Fig. 6:**
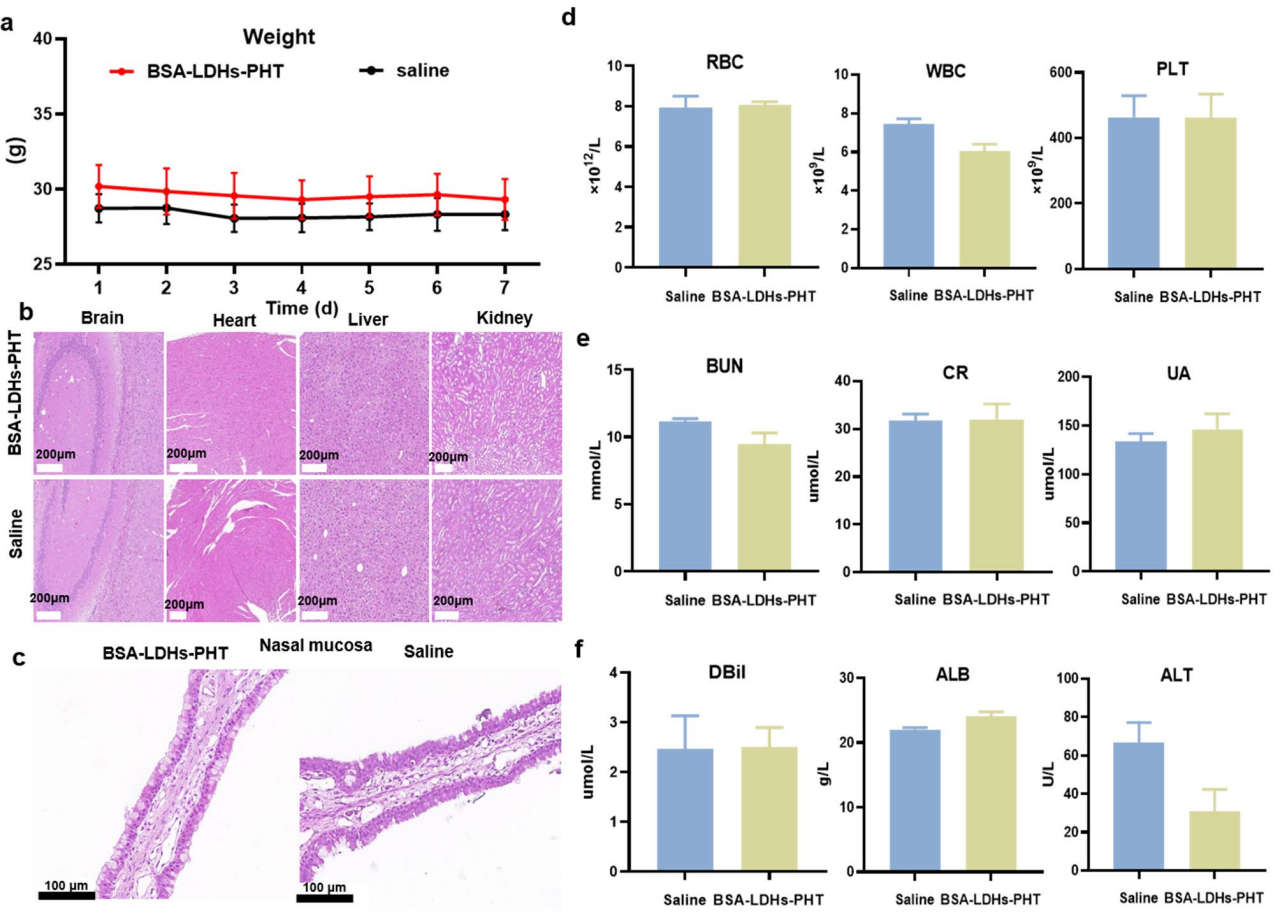
Toxicity assessment of BSA-LDHs-PHT. **a** Daily weight changes during administration in mice. **b, c** Histopathological evaluation of major organs (brain, heart, liver, and kidneys) **(b)** and mice nasal mucosa **(c)** was performed after a 7-day repeated dose study. **d** Blood routine (RBC, WBC, PLT) of two groups of mice after 7 days of administration. **e** Renal function (blood urea nitrogen, creatinine, uric acid) of two groups of mice after 7 days of administration. **f** Liver function (Direct bilirubin, albumin, ALT) of two groups of mice after 7 days of administration. Data expressed as mean ± sem (*n* = 4). * *p* < 0.05; ** *p* < 0.01; *** *p* < 0.001

According to previous literature reports, LDHs had minimal impact on systemic growth in Sprague-Dawley rats at doses up to 200 mg/kg [51]. As shown in the Fig. 6a, no significant weight changes were observed in both groups of mice. To evaluate the local and systemic safety of BSA-LDHs-PHT, we conducted a histopathological assessment of nasal mucosa and major organs (brain, heart, liver, and kidney) in mice following repeated administration for 7 days. Representative histologic sections of nasal mucosa and solid organs are presented in Fig. 6b, c. After continuous administration for 7 days, the BSA-LDHs-PHT group did not exhibit nasal mucosal damage or induce separation between the pseudocomplex columnar epithelium and basement membrane when compared with the negative control group (0.9% NaCl). Regarding systemic toxicity, no histopathological alterations were observed in mice’s brain, heart, liver, and kidneys across all groups. Hematological parameters of blood routine (RBC, WBC, PLT), renal function (blood urea nitrogen, uric acid, creatinine), and liver function (direct bilirubin, albumin, ALT) of each group were all at a normal level (Fig. 6d, e, f), verifying the negligible impact on blood indicators. The results demonstrated that BSA-LDHs-PHT exhibited favorable biocompatibility with both the nasal cavity and major organs, thereby establishing its potential as a non-toxic nasal formulation for treating patients with epilepsy.

## Conclusions

In this study, we prepared a nanomedicine, BSA-LDHs-PHT, for epileptic seizure control via I.N. administration. The BSA-LDHs-PHT has an initial explosive release and subsequent slow-release characteristics. In vivo fluorescence imaging and PTZ-induced acute seizure model demonstrated good brain-targeted release and high therapeutic effect of BSA-LDHs-PHT after I.N. delivery. No signs of toxicity were observed in peripheral organs. Therefore, the I.N. delivery of BSA-LDHs-PHT improves drug utilization and ensures the organism’s safety, which provides a promising approach to ensure the effective treatment of epilepsy. In addition to phenytoin, combining nano drug delivery systems with I.N. drug delivery will bring new possibilities to more ASMs in the future.

### Electronic supplementary material

Below is the link to the electronic supplementary material.

Supplementary Material 1

## Data Availability

The data for this study can be obtained from the corresponding author according to reasonable requirements.

## Abbreviations

PHT: phenytoin
BBB: blood-brain barrier
H&E: hematoxylin and eosin
I.N.: intranasal
ASMs: antiseizure medications
LDH nanoparticles: layered double hydroxide nanoparticles
BSA: bovine serum albumin
BSA-LDHs: combining the LDH nanoparticles with bovine serum albumin
BSA-LDHs-PHT: BSA-LDH nanoparticles loading with phenytoin
Cy5.5: Cyanine5.5
FTIR: fourier transform infrared
