# Supplementary material for: Intranasal delivery of phenytoin loaded layered double hydroxide nanoparticles improves therapeutic effect on epileptic seizures
Source: J Nanobiotechnology. 2024 Apr 2;22:144. doi: 10.1186/s12951-024-02405-8 (PMC10985904; doi:10.1186/s12951-024-02405-8)
Supplement: Supplementary file 1 — Supplementary Material 1 [file 12951_2024_2405_MOESM1_ESM.docx]

**Intranasal delivery of phenytoin loaded layered double hydroxide nanoparticles improves therapeutic effect on epileptic seizures**

**This PDF file includes:**

Fig. S1 to


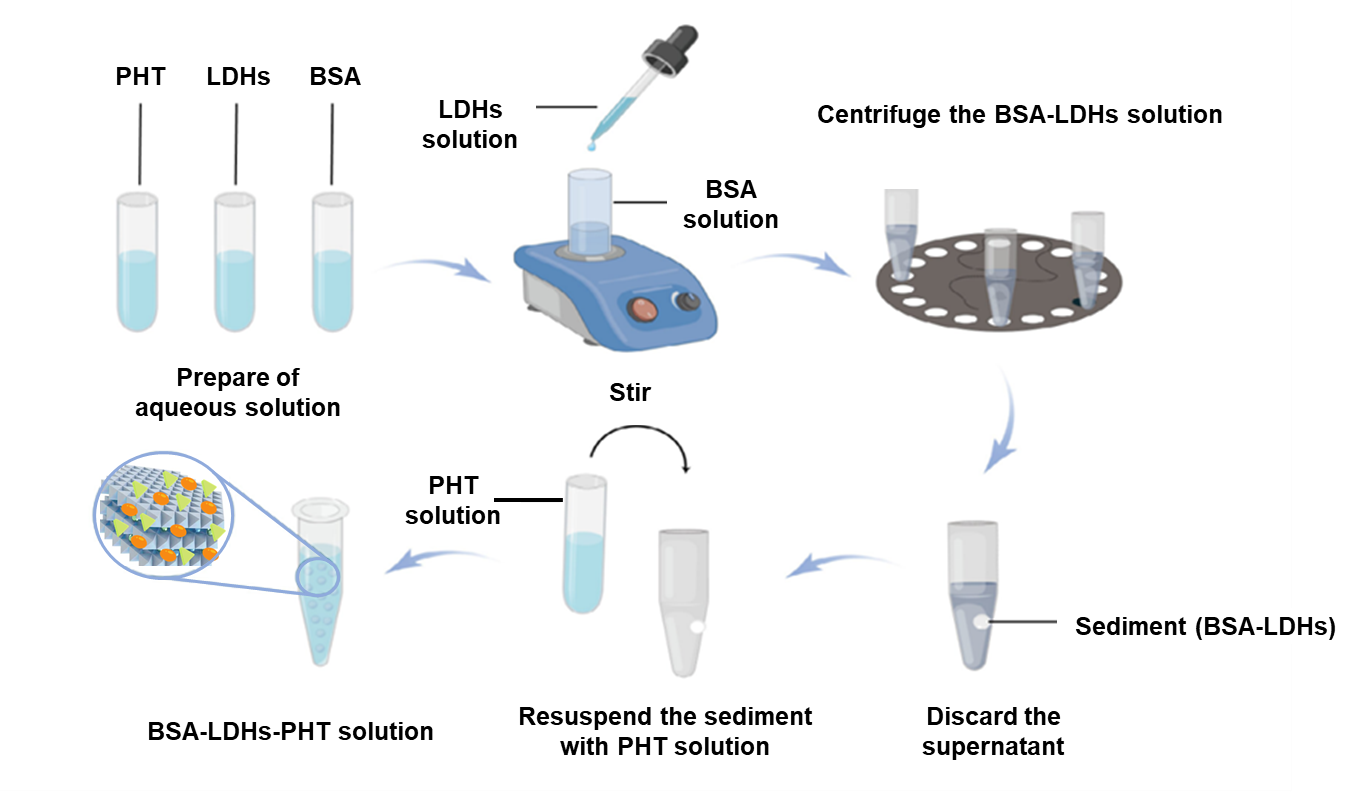


**Supplementary Fig. 1** Schematic representation of the method of preparation of BSA-LDHs-PHT

Fig. S2 to


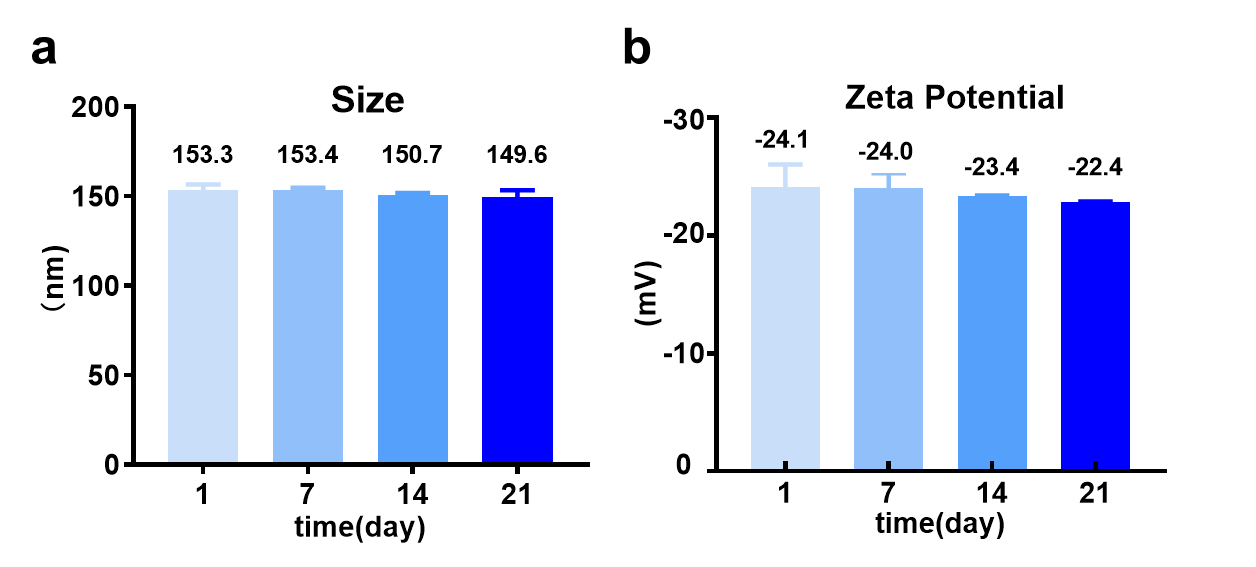


**Supplementary Fig. 2** The size **(a)** and zeta potential **(b)** of BSA-LDHs-PHT within 21 days, p$\text{<}\text{ }$0.05


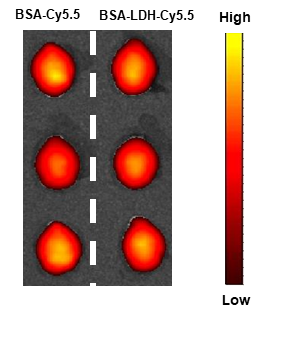
Fig. S3 to

**Supplementary Fig. 3** In vitro fluorescence images of mice brains after 15 minutes of administration (n=3)

Fig. S4 to


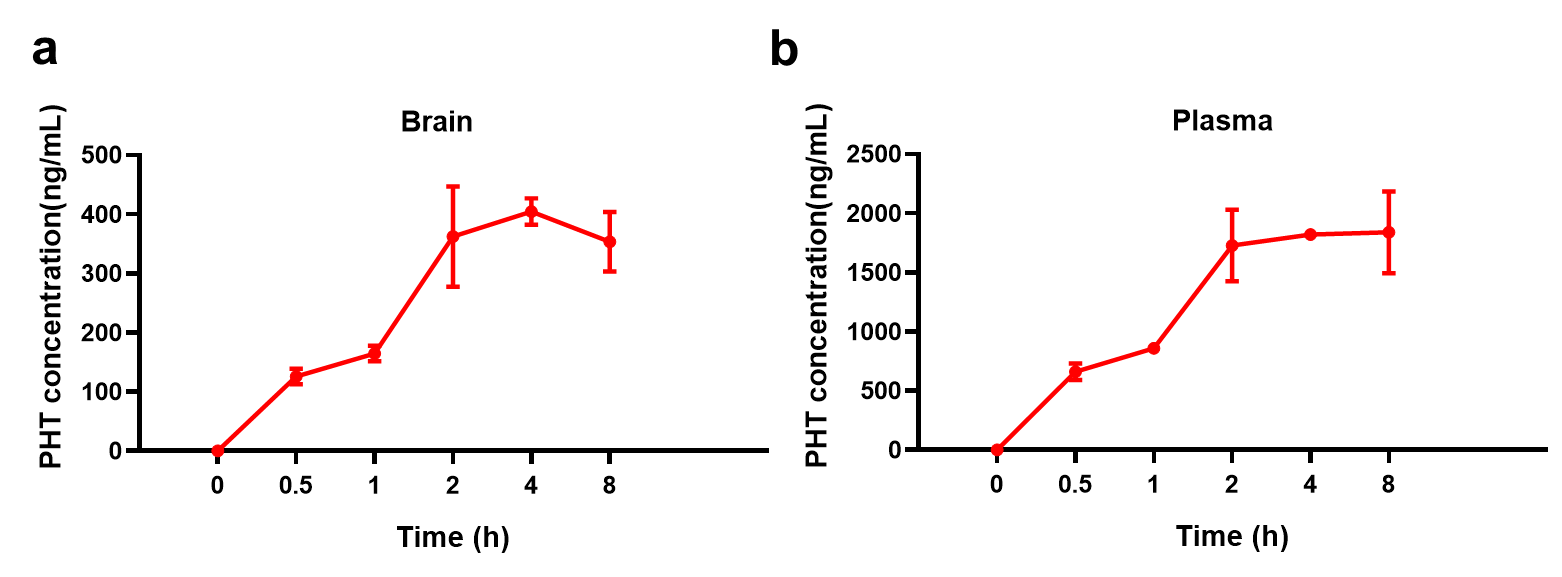


**Supplementary Fig. 4** Concentration-time profiles of PHT (n=3) in brain **(a)** and plasma **(b). a** The C_max_ of phenytoin in brain is 404.3 ng/mL at 4 hours. **b** The C_max_ of phenytoin in plasma is 1840 ng/mL at 8 hours.

Table S1 to

**Supplementary Table 1.** Study design for in vivo anti-seizure effects study

| **Group** | **Total No. of animals** | **Animal subgroups based on different durations after administration** | | **Treatment** | **Dose-volume** | **Drug amount** |
| --- | --- | --- | --- | --- | --- | --- |
|  |  | **5 mins** | **30 mins** |  |  |  |
| 1 | 20 | 10 | 10 | I.N. (Saline) | (25μL/nostril)*2 | 200 μg |
| 2 | 20 | 10 | 10 | Oral (PHT) | (25μL/nostril)*2 | 200 μg |
| 3 | 20 | 10 | 10 | I.N. (PHT) | (25μL/nostril)*2 | 200 μg |
| 4 | 20 | 10 | 10 | I.N. (BSA-LDHs-PHT) | 200 μL | 200 μg |
